# Intensive short-term vasodilation effect in the pain area of sciatica patients - case study

**DOI:** 10.1186/1756-0500-7-620

**Published:** 2014-09-09

**Authors:** Elżbieta Skorupska, Michał Rychlik, Wiktoria Pawelec, Agata Bednarek, Włodzimierz Samborski

**Affiliations:** Department of Rheumatology and Rehabilitation, Poznan University of Medical Sciences, Poznan, Poland; Department of Virtual Engineering, Poznan University of Technology, Poznan, Poland; Department of Biomechanics, University School of Physical Education, Poznan, Poland

**Keywords:** Sciatica, Trigger points, Infrared camera, Vasodilation, Autonomic phenomenon

## Abstract

**Background:**

Varied and complicated etiology of low back pain radiating distally to the extremities is still causing disagreement and controversy around the issue of its diagnosis and treatment. Most clinicians believe that the source of that pain is generally radicular. While some of them postulate the clinical significance of the sacroiliac joint syndrome, others demonstrate that almost one in five people with back pain experience symptoms indicative of the neuropathic pain component. To date, neuropathic involvement has not been completely understood, and different mechanisms are thought to play an important role. It has been established that muscle pain (myofascial pain) e.g. active trigger points from the gluteus minimus, can mimic pain similar to sciatica, especially in the chronic stage. This paper describes patients presenting with radicular sciatica (case one and two) and sciatica-like symptoms (case three). For the first time, intensive short-term vasodilation in the pain area following needle infiltration of the gluteus minimus trigger point was recorded.

**Case presentation:**

Three Caucasian, European women suffering from radicular sciatica (case one and two) and sciatica-like symptoms (case three) at the age of 57, 49 and 47 respectively underwent infrared camera observation during needle infiltration of the gluteus minimus trigger point. The patients were diagnosed by a neurologist; they underwent magnetic resonance imaging, electromyography, neurography and blood test analysis. Apart from that, the patients were diagnosed by a clinician specializing in myofascial pain diagnosis.

**Conclusion:**

In the examined cases, trigger points-related short-term vasodilation was recorded. Confirmation of these findings in a controlled, blinded study would indicate the existence of a link between the pain of sciatica patients (radicular or sciatica-like pain) and the activity of the autonomic nervous system. Further studies on a bigger group of patients are still needed.

**Electronic supplementary material:**

The online version of this article (doi:10.1186/1756-0500-7-620) contains supplementary material, which is available to authorized users.

## Background

Varied and complicated etiology of low back pain radiating distally to the extremities is still causing disagreement and controversies around the issue of its diagnosis and treatment. Most clinicians believe that the source of that pain is generally radicular. While some of them postulate the clinical significance of the sacroiliac joint syndrome [1], others demonstrate that almost one in five people with back pain experience symptoms indicative of the neuropathic pain component. To date, neuropathic involvement has not been completely understood, and different mechanisms are thought to play an important role. A combination of nociceptive and neuropathic pain-generating mechanism is thought to be involved, which led to the establishment of the term mixed pain syndrome [2]. It has been established that muscle pain (myofascial pain) e.g. active trigger points from the gluteus minimus, can mimic pain similar to sciatica, especially in the chronic stage [3]. Trigger points (TrPs) are hyperirritable nodules within taut bands of skeletal muscles, which can provoke sensory, motor, and autonomic symptoms. These symptoms can be released through the damage of disturbed neuromuscular junction (trigger point) by rapid, repetitive needle insertion to the trigger points called dry needling (DN), which causes muscle injury and may damage nerve fibers [4, 5].

The diagnosis of myofascial pain (MPS) is based on palpatory criteria defined by Simons and Travell [3]. Even though MPS diagnostic criteria have been precisely defined, some clinicians are still denying their existence. Nowadays, a rapid development of basic knowledge of MPS and its diagnosis is observed. As an example, trigger points have been objectively verified by means of magnetic resonance imaging [6, 7] sonoelastography [8] or intramuscular electromyography [9]. Unfortunately, these techniques are not easily applicable to clinical practice at this time. For that reason, the diagnosis of MPS is still based on palpatory diagnostic criteria.

This paper describes patients presenting with chronic low back pain radiating to the left lower limb diagnosed neurologically as radicular sciatica (case one and two) and sciatica-like symptoms (case three). Additionally, patients were re-diagnosed with myofascial pain syndrome by another clinician. The examined patients presented autonomic nervous system activity, namely intensive short-term vasodilation effect in the pain area after needling infiltration of the gluteus minimus trigger point.

## Case presentation

### Ethics Statement

The study was conducted in accordance with the Declaration of Helsinki approved by the Ethics Committee of Poznan University of Medical Sciences (no. 630/13). All subjects gave written informed consent to participate in the study before data collection. A detailed description of all examination and treatment procedures, including dry needling (DN), and risks involved in this study has been provided to the participants. Participants had the right to refuse DN treatment and withdraw from the study at any time without penalty.

### Case 1

A 57-year-old female presented to the University outpatient clinic with a twelve weeks history of progressive pain in the left lower extremity and low back pain (LBP). Her past medical history was remarkable for several prior episodes of lower back pain. She described her state as aggravating with time, her pain ranged between 3 and 8 points on the visual-analogue scale (VAS). The patient reported pain in the lower back, groin and posterolateral thigh. She complained of some temporary but severe pain in the calf and foot, especially during the night. Standard treatment for radicular pain failed (she received miorelaxants, anti-inflammatory drugs and pain-killers (paracetamol, opioids). She was re-diagnosed with sciatica of radicular origin by an independent university neurologist. The diagnosis was based on detailed bedside neurological examinations and extensive neurological screening examination (NSE). Lasegue test, femoral nerve tension test, passive dorsiflexion test (Bragard test) and Lasegue test plus neck flexion were all positive, cross Lasegue sign was the only negative.

The locomotor system examination confirmed tension of the paraspinal muscles on the left side at L2/L3 and pain of the spinous processes. Although a slight muscle weakness for flexion and extension (4/5) of the thigh and knee on the right side was discovered, other parameters were normal (5/5). Results of other neurological assessments were normal. Magnetic resonance imaging (MRI) scan of the lumbar and sacral spine revealed right disc protrusion at L3-L4 indenting thecal sac and the same side disc-root conflict. No abnormalities in sacroiliac joint were found. In the electromyographical examination, (erector spinae, quadratus lumborum, gluteus maximus, rectus femoris and gastrocnemius), a normal result was found during rest. In maximum contraction, features of bilateral motor unit dysfunction in innervation of L4-L5, L5-S1 were discovered. Conduction of motor fibers in examined nerves in lower limbs was normal, as was conduction of motor fibers in ventral root L5-S1. Laboratory tests revealed leukopenia, neutropenia, lymphopenia and monocytosis (WBC 3,3×10µ3/ µL, 1,6×10µ3/ µL, 1,3 ×10µ3/ µL and 10,9% respectively, ESR and C-reactive protein (CRP) were within limits ESR -12/lh, CRP -0.1 mg/l.

### Case 2

A 49-year-old female presented to the University outpatient clinic with a four weeks history of intensive pain in left lower extremity and low back pain. Medical history revealed several prior episodes of LBP which started in 2003. The patient confirmed the level of pain as 8 according to VAS. The pain covered her lower back, lateral thigh, calf and lateral edge of the foot. Apart from the pain, she also complained of numbness. Previous sciatica treatment failed (she received thiamin pyrophosphate, cobalamin, anti-inflammatory drugs and paracetamol). Based on detailed bedside neurological examinations and NSE, she was re-diagnosed with sciatica of radicular origin by an independent university neurologist. These examinations revealed abnormal gait on the heel of the left foot, slight weakness of the left foot inversion (4/5), left foot dorsiflexion (4/5), great toe dorsiflexion (4/5) and temperature dysesthesia of the lateral edge of the foot. While the examination revealed a positive Lasegue test, Bragard test and Lasegue test plus neck flexion, the rest of the tests were negative. Other results of neurological examination were normal. The following results were confirmed through MRI examination: posterior disc bulging without thecal sac compression at L2-L3, minor disc protrusion with thecal sac modelling and a disc-root conflict at L3-L4, bilateral disc-root conflict at L5, severe compression of the left nerve root and thecal sac at L5-S1, and L5-S1 disc space narrowing due to disc protrusion. No abnormalities in sacroiliac joints were found. Similarly to case one patient, electromyographic and electroneurographic examinations were carried out. Although their results turned out to be normal, during maximuml contraction, motor unit dysfunction features were discovered in the left side of L4-L5 innervation. Laboratory tests revealed a slight monocytosis (11%), elevated MCV (116.3 fL) and MCH (39.5 pg). Other parameters were normal - the erythrocyte sedimentation rate (ESR) reached 4/lh, C-reactive protein levels (CRP) reached 0.1 mg/l.

### Case three

A 46-year-old female presented to the University outpatient clinic with a three weeks history of intensive pain in the left lower extremity and low back pain. She confirmed the level of pain on five according to VAS. Patient localised the pain in the lower back, posterior-lateral thigh and calf. She received pain killers and anti-inflammation drugs from her GP. She was diagnosed by university neurologist with sciatica-like symptoms on the basis of bedside examinations and NSE (a norm for every component of the test was confirmed). Leftward disc herniation at the L5-S1 with left nerve root intracanal compression were the only abnormalities registered in MRI. In electroneurography, no dysfunction of peripheral conduction of lower limb nerves was discovered. However, by means of electromyography, features of motor unit dysfunction in L4-L5 innervation were recorded on the left side during maximal contraction. Laboratory tests confirmed neutropenia (NEUT#1.93×10µ3/ µL; NEUT% 33.6%), slight monocytosis (MO# 0.83×10µ3/ µL; MO% 14.3%); elevated levels of lymphocytes 49.8%; and trombocytosis 863×10µ3/ µL. Other results were normal - ECR 8/lh, CRP 5.3 mg/l.

### Myofascial pain examinations

In the patients, the presence of an active trigger point in the anterior fibres of the gluteus minimus muscle (GM) was confirmed according to Travell and Simons’ criteria [3]. Within the GM, two most painful points with referred pain were marked. Then, the participants were subject to dry needling technique performed in the previously marked points under control of an infrared thermovision camera. Needling was performed with 0.30 mm diameter, 60 mm long sterile acupuncture needles SE L (Serin Corp, Shizuoka, Japan). Each needle was packed separately. The needle pierced the skin and reached the painful point with referred pain. The recognizable pain started to withdraw partially and repeatedly and eventually both the pain and muscle fiber contraction subsided. The time of needling was maximum 6 minutes for any given point. After needling of both points was conducted, further thermovisual imaging was performed. At the end, a set of thermovisual images of the final state was recorded. For the diagnostic test, a thermovision touchless camera using the 8-14 µm waveband and working in real time was applied. The camera was equipped with uncooled VOx (vanadium oxide) microbolometer. To obtain the stability of patient’s body temperature and to ensure the adjustment of the recording camera’s temperature to the interior conditions, the evaluation began 30 minutes after the patient had entered the examination room. Thermal isolation of the evaluated area from other thermal factors that might have influenced the evaluation, including other parts of the patient’s and doctor’s bodies was ensured. Moreover, when performing thermovisual imaging, the general rules of camera usage were followed.

During the evaluation, all thermovisual images were measured and recorded within the measurement range displayed by the camera and set between 25 and 45 degrees Celsius (in individual cases lower levels of temperature can be set).

### Results of the infrared thermovisual observation

**Figure 1:**
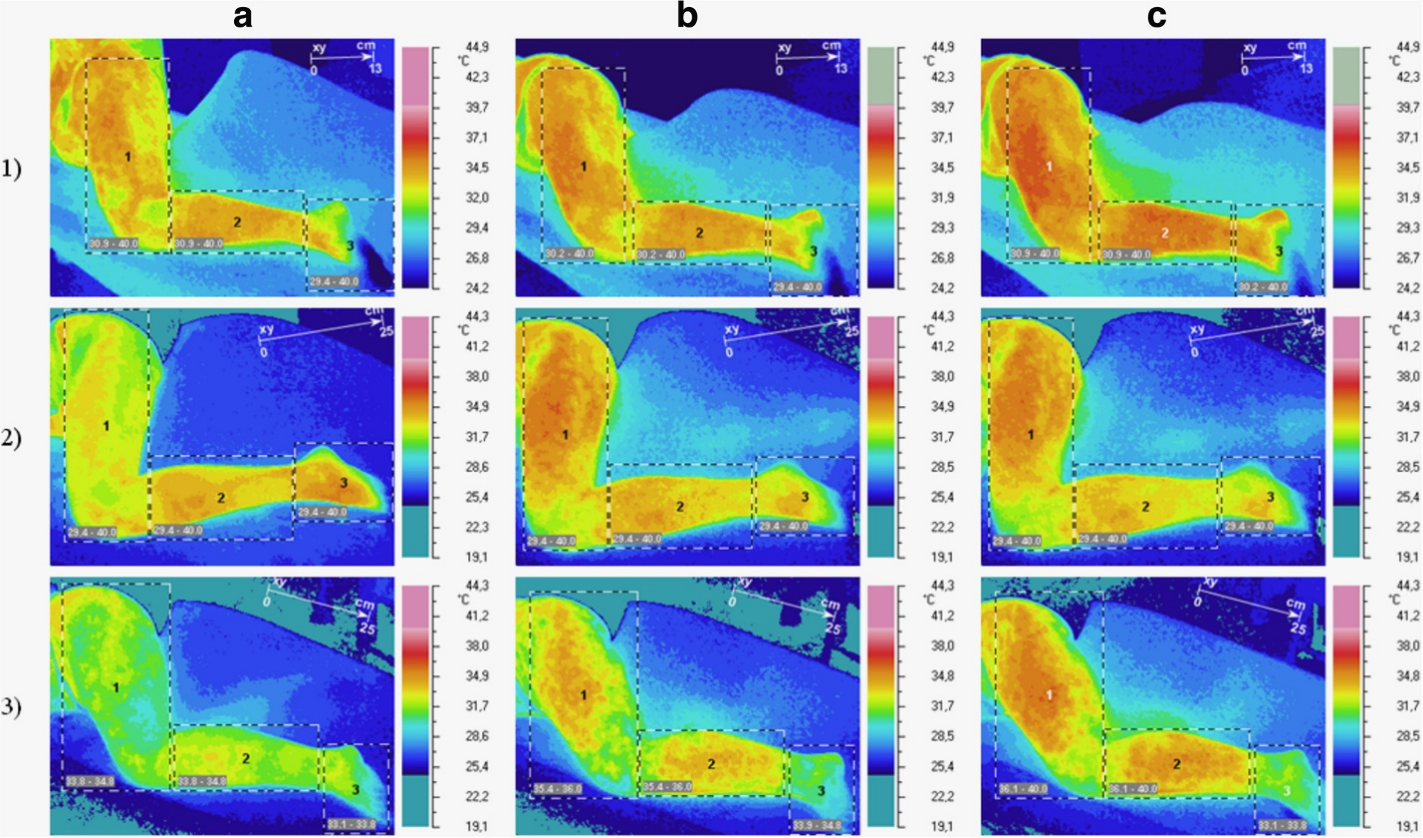
**Temperature reaction measured on skin surface (short-term vasodilation effect).** In rows, cases one, two and three have been shown. In columns, pre-DN state has been marked with **(a)**, state immediately post-DN has been marked with **(b)**, and post-needling observation has been marked with **(c)**.

**Figure 2:**
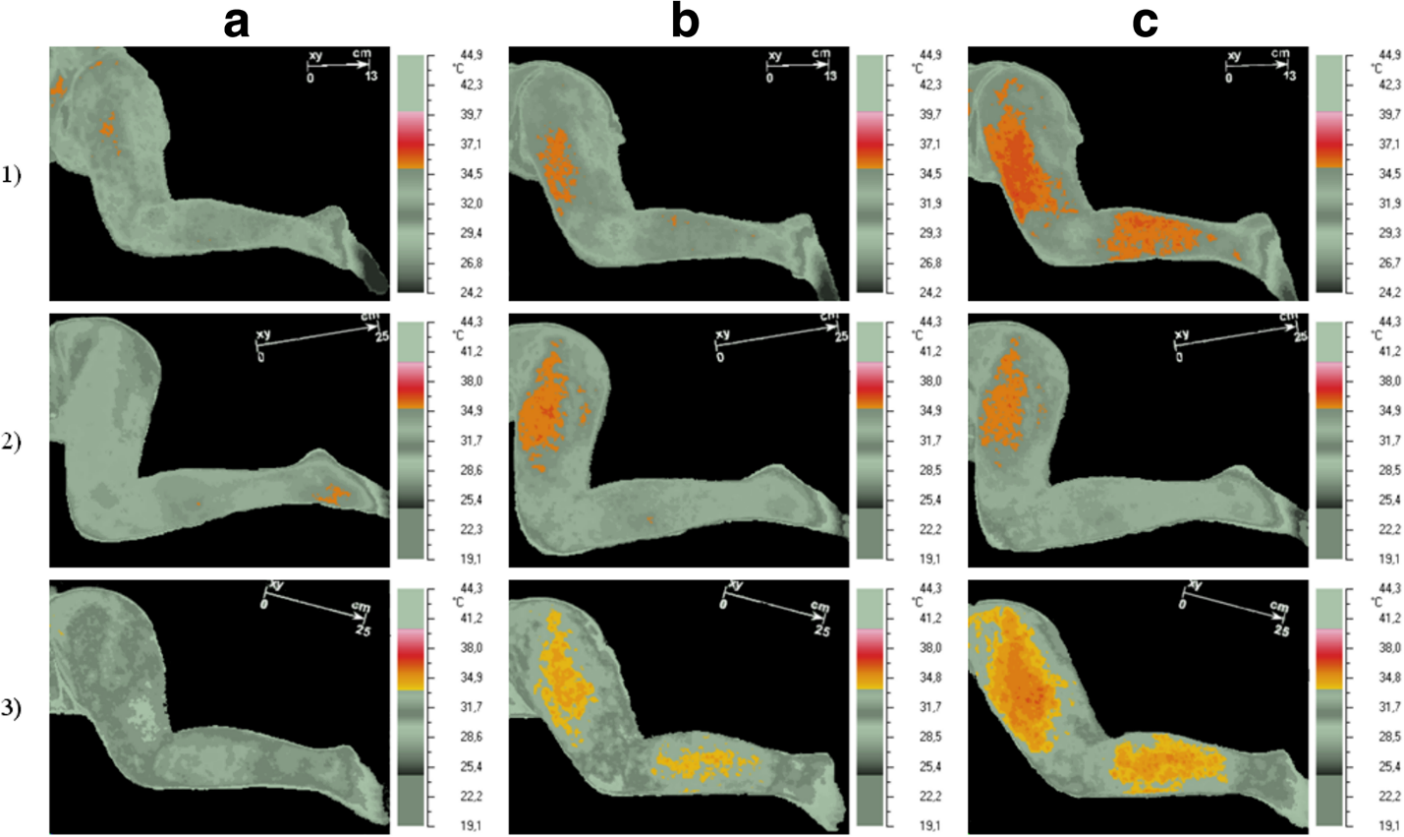
**Visualization of the highest temperature reaction measured on the skin surface (short-term vasodilation effect).** In order to depict the area of vasodilation, pre-DN patient temperature reaction has been isolated – grey pictures column **(a)**. In column **(b)** and **(c)**, the area of vasodilation higher than Tmax of the initial state is presented.

After dry needling of active TrPs in the gluteus minimus muscle in the pain area (sciatica), the short-term vasodilation effect was recorded (under infrared thermovision camera) (Figure 1). The increase of maximum and average temperature (Tmax, Tavr) limited to the subarea with vasodilation effect was confirmed (case 1 ΔT max +1.3°C; ΔT avr 0.9°C; case 2 ΔT max +2.1°C; ΔT avr 1.1°C; case 3 ΔT max +2.1°C; ΔT avr +1.8°C). Subareas with the vasodilation of the highest temperature increase are shown on Figure 2. Dry needling provoked spreading of subareas (iso-areas) with the highest temperature. For case one patient, the iso-area 6.9 cm^2^ (35.6°C) spread to 59.9 cm^2^ (35.6°C -36.2°C) post-needling, and in rest position six minutes later, further spreading was observed – finally the area reached 361.4 cm^2^ and the temperature rose to 36.9°C, then a slow decrease of the vasodilation subarea and temperature were observed. For case two respectively: 9.1 cm^2^ (35.7°C) to 119.3 cm^2^ (36.2°C) to 232.3 cm^2^ (36.2°C). For case three: 0.1 cm^2^ (34.2°C) to 508.6 cm^2^ (34.9°C) to 635.7 cm^2^ (36.5°C).

In each of these cases, the first manifestations of vasodilation were recorded immediately after needle insertion to a trigger point. The vasodilation effect increased during DN, reached its highest at approximately 6 minutes post DN and decreased afterwards. These reactions depended on pain severity during procedure and the time post-DN (Additional file 1).

## Discussion

The main outcome of this study was to establish the short-term vasodilation effect in the pain area of patients diagnosed with sciatica. The reaction recorded during procedure may indicate the existence of a link between sciatica and sympathetic nerve activity, which has already been suggested [2]. Additionally, the involvement of myofascial pain syndrome among patients diagnosed with radicular or sciatica-like symptoms is possible. Although the findings presented in this paper are highly unique, the existence of autonomic phenomena (e.g. vasoconstriction in the area to which a trigger point refers pain) has already been suggested by Travell and Simons [3]. Some authors proved that intramuscular needling can induce vasodilation, but only locally [10]. Others tried to confirm the idea of Travell and Simons and confirmed the activation of the autonomic nervous system after nociceptive stimulation of latent TrPs (glutamate injections) in a non-symptomatic subject. However, they found IRT unable to detect vasodilation after TrP stimulation and they preferred to measure skin blood flow by laser Doppler flowmetry [11, 12]. Quite interestingly, in other studies, the needle stimulation of the latent trigger point, skin and muscle point (acupuncture) provoked a decrease of skin temperature contrary to observations in this study [11–13]. In the observations presented in this study, the vasodilation effect increased during DN, reached its highest post-DN and decreased afterwards. The vasodilation within the skin is provoked by Calcitonin Gen Related Peptide (CGRP) and Substance P release from Aδ nerve fibers and/or C-fibers [5, 14, 15]. Moreover, it is suggested that CGRP-related antidromic vasodilation may be important in clinical improvement of skeletal muscle blood flow produced by physical therapy, e.g. acupuncture [16]. Although the exact mechanism of DN remains unexplained, some authors propose that DN of active trigger points can stimulate sensory afferent Aδ nerve fibers [5]. In the light of findings presented in this study, it seems that trigger point needle stimulation probably activated the sympathetic vasoconstriction mechanism and antagonized the primary afferent nerve-induced antidromic vasodilation. As mentioned above, some authors postulated the idea of the autonomic nervous system activity involvement among patients suffering from sciatica [2]. It seems that the cases presented in this paper support that idea. Although clinical signs of examined patients during bedside examination were typical of sciatica, the MRI results can spark some controversy. In the case of the patient with sciatica-like symptoms (case three), a disc-radicular conflict was found on the affected side. In the case of the patient with radicular pain diagnosis (case one), the MRI revealed a disc-radicular conflict on the opposite side (cross Lasegue was negative). Only in the case of patient two, both bedside examination and MRI results were typical of her condition. Nevertheless, no changes in nerve conduction or spinal reflex pathologies were found in any of the patients. It is now postulated that traditional diagnosis of sciatica patients should be followed by additional examinations, especially due to the fact that diagnostic accuracy of neurological signs and tests is unclear according to the literature on the subject [17]. Lasegue test is the only sign consistently reported to be sensitive for sciatica due to disc herniation, but its credibility is limited by its low specificity. Similarly, in MRI analysis, despite the fact that in majority of patients with pathology within disc area a strong correlation with pain in the lower limb is visible [18], sometimes it is possible to observe improvement with no changes concerning the disk [19], or the other way round: no improvement in spite of removing the disc protrusion or other reasons of nerve compression [20]. What is more, in some asymptomatic individuals, herniated nucleus pulposus occurred in MRI. There are also reports of patients suffering from confirmed disk pathology or with stenosis with apparent neural compromise, i.e. asymptomatic [21–23]. Apart from that, the laboratory blood test did not show any sign of inflammation. The situation in which laboratory blood tests, radiologic investigation or bedside examinations could not explain the source of the pain is typical of the neuropathic pain component. Because the examined patients did not undergo diagnostic tests evaluating neuropathic pain, it has not been proven that they suffered from neuropathic pain. However, in the light of findings presented in this study and existing theories stating that neuropathic pain patients suffer because of the activation of muscle fibers by sympathetic nerve activity [24], in the future it is important to evaluate the link between sciatica, neuropathic pain and trigger points. According to Travell, myofascial pain trigger points can mimic sciatica [3]. Myofascial pain and active trigger points of the gluteus minimus muscle were the only features to be found in all of the three examined cases. The aim of the study was not to deny the neurological diagnosis, however, clinical value of trigger points should be explored in the future. Further studies on a bigger group of sciatica patients co-diagnosed with myofascial pain are needed.

## Conclusion

Conclusion: in the examined cases, trigger points related short-term vasodilation was recorded. Confirmation of these findings in a controlled, blinded study would indicate the existence of a link between the pain of sciatica patients (radicular or sciatica-like pain) and the activity of the autonomic nervous system. Further studies on a bigger group of patients are still needed.

## Consent

Written informed consent was obtained from the three patients for publication of this case report and any accompanying images. A copy of the written consents are available for review by the Editor of this journal

## Electronic supplementary material

Additional file 1: **Short-term vasodilation effect during DN completion and observation time (case one).** The first three seconds picture the state of the patient pre-DN. In the third second, the DN of the first TrP can be observed. In the eighth second, the end of DN of the first TrP can be seen. In the eleventh second of the footage, the end of DN of the second TrP has been recorded. The final part of the footage (observation time, starting from eleventh and lasting until the fifteenth second) presents post-DN increase of vasodilation. (AVI 17 MB)

## Abbreviations

CGRP: Calcitonin Gen Related Peptide
CRP: C-reactive protein
DN: Dry needling
ESR: Erythrocyte sedimentation rate
GM: Gluteus minimus muscle
IRT: Infrared thermovision camera
LBP: Low back pain
MPS: Myofascial pain
MRI: Magnetic resonance imaging
NSE: Extensive neurological screening examination
Tavr: Average temperature
Tmax: Maximum temperature
TrPs: Trigger points
VAS: Visual-analogue scale.
